# Maternal supplementation with *Bacillus*-based direct-fed microbials altered the maternal and offspring fecal microbiome

**DOI:** 10.1093/tas/txaf145

**Published:** 2025-10-31

**Authors:** Luciana M Sousa, Vinicius S Izquierdo, Bruno I Cappellozza, Philipe Moriel

**Affiliations:** Range Cattle Research and Education Center, IFAS/University of Florida, Ona, FL 33865, United States; Range Cattle Research and Education Center, IFAS/University of Florida, Ona, FL 33865, United States; Novonesis, Lyngby 2800, Denmark; Range Cattle Research and Education Center, IFAS/University of Florida, Ona, FL 33865, United States

**Keywords:** *Bacillus*, beef cattle, *Bos indicus*, microbiome

## Abstract

This study evaluated the effects of maternal supplementation of a *Bacillus*-based direct-fed microbial (DFM) on fecal microbiome of heifer-calf pairs. At the start of the study (day 0), 72 pregnant Brangus crossbred beef heifers (20 to 22 mo of age) were stratified by body weight (BW; 431 ± 31 kg) and body condition score (BCS; 6.0 ± 0.36) and randomly allocated into 1 of 12 bahiagrass pastures (1 ha and 6 heifers/pasture). Treatments were assigned to pastures and consisted of heifers supplemented with 1 kg/hd/d of soybean hulls added (BAC) or not (CON) with DFM containing *Bacillus subtilis* 810 and *B. licheniformis* 809 (3 g/hd/d; 6.6 × 10^9^ colony forming unit; Bovacillus; Novonesis, Lyngby, Denmark) from day 0 to 242 (139 ± 4 d prepartum to 104 ± 4 d postpartum). Calves were early weaned on day 242 and then allocated to drylot pens and fed the same diet until day 319. On days 271 and 287, calves were vaccinated against pathogens associated with bovine respiratory disease. Fecal samples were collected from 3 heifers per pasture on days 0, 90 and 180 and from 2 to 3 calves per pen on days 242 and 272. Shannon and Simpson diversity indexes tended to be greater (*P *= 0.09) for BAC vs. CON heifers. *Clostridium* and *Blautia* relative abundances on day 90 and average *Mogibacterium* relative abundance were lower (*P *≤ 0.03) for BAC vs. CON heifers, whereas *Bacteroides* and *Porphyromonas* relative abundances tended (*P *≤ 0.08) to be greater for BAC vs. CON heifers. Shannon diversity index did not differ (*P *≥ 0.14) between CON and BAC calves, whereas Simpson diversity index remained constant (*P *= 0.98) for CON calves from day 242 to 272 but increased (*P *= 0.02) for BAC calves from day 242 to 272. Effects of maternal treatment × day tended (*P *= 0.06) to be detected for *Paraprevotella* genus, which *Paraprevotella* relative abundance on day 242 was lower (*P *= 0.05) for BAC vs. CON calves on day 242, and did not differ (*P *= 0.89) between treatments on day 272. Relative abundance of *Bacteroides* was greater (*P *= 0.01), whereas *Slackia* was lower (*P *< 0.01) for BAC vs. CON calves. *Blautia, Butyrivibrio*, and *Methanobrevibacter* relative abundance tended (*P *= 0.08) to be lower for BAC vs. CON calves. In conclusion, exclusive maternal supplementation with a *Bacillus*-based DFM during gestation and early lactation modulated the fecal microbiota of both heifers and their offspring.

## Teaser

Maternal exclusive supplementation with *Bacillus*-based direct-fed microbials during prepartum and postpartum modulated both maternal and offspring fecal microbiome.

## Introduction

The inclusion of direct-fed microbials (DFM) in the nutrition of dairy and beef cattle has gained focus as a strategy to enhance ruminal and intestinal function and as a natural alternative to antibiotics (Cappellozza et al. 2025). Among the DFM, *Bacillus* spp. has shown potential to stimulate ruminal fermentation (Ushakova et al. 2013) and improve animal health and performance by inhibiting potentially harmful bacteria (Capern et al. 2025; McAllister et al. 2011) and enhancing gut integrity (Boll et al. 2024; Copani et al. 2020; Santano et al. 2020). Maternal nutrition during gestation, including DFM supplementation, is known to influence offspring growth and vaccine-induced immune responses (Izquierdo et al., 2023, 2024; Moriel et al. 2021, 2024, 2025). For instance, exclusive maternal supplementation with a *Bacillus*-based DFM during prepartum and postpartum increased prepartum body condition score (BCS) of beef heifers and enhanced the post-weaning growth and humoral immune response of their calves compared with non-supplemented heifers (Izquierdo et al. 2024). Although the underlying mechanisms influencing these ­outcomes in offspring performance remain unclear, potential factors include modifications to both the maternal and offspring metabolome (Izquierdo et al. 2025) and, perhaps, gut microbiome.

The gastrointestinal tract serves as a habitat for a diverse and dynamic bacterial community that can affect growth and health of the host, with early-life gut colonization shaping the gut environment and long-term performance of the host (Rodríguez et al. 2015). One of the most important factors influencing the gut microbiome is the type and amount of nutrients consumed by the animal (Kim et al. 2014; Myer et al. 2015), including DFM (Cappellozza et al. 2025). Studies investigating the effects of maternal supplementation with *Bacillus*-based DFM on the gut microbiome of cow-calf pairs are lacking. Understanding the composition of the fecal microbiota is important not only for mitigating pathogens through dietary changes, but also for better understanding how maternal nutrition influences offspring microbial development. We hypothesized that an exclusive maternal supplementation with a *Bacillus*-based DFM during prepartum and postpartum periods will influence both the maternal and offspring fecal microbiome, partially influencing the previously observed improvements in calf post-weaning growth and immune response (Izquierdo et al. 2024) and altered calf plasma metabolome (Izquierdo et al. 2025). Thus, our objectives were to evaluate the fecal microbiome of both heifers and their offspring following the exclusive maternal supplementation with a *Bacillus*-based DFM during the prepartum and early post-partum periods.

## Materials and methods

The experiment (Izquierdo et al. 2024) was conducted at the University of Florida, Institute of Food and Agricultural Sciences, Range Cattle Research and Education Center, Ona, FL (27°23ʹN and 81°56ʹW) from May 2022 to November 2023. All animals used in this experiment were cared for by practices approved by the University of Florida—Institute of Animal Care and Use Committee (protocol #202111585).

### Animals and diets

#### Maternal management

Further details on the management and data collection in both pregnant heifers and their calves were described previously by Izquierdo et al. (2024). On day 0 of the study (∼139 d before calving), 72 pregnant, Brangus crossbred heifers (20 to 22 mo of age) were stratified by their initial body weight (BW= 431 ± 31 kg) and body condition score (BCS = 6.0 ± 0.36; scale 1 to 9), and then assigned to 12 bahiagrass (*Paspalum notatum)* pastures (6 heifers and 1 ha per pasture) in a completely randomized design, where each pasture represented an experimental unit. Maternal treatments were randomly assigned to pastures (6 pastures per maternal treatment) and consisted of heifers supplemented with 1 kg per head daily [dry matter (DM) basis] of loose soybean hulls, either alone (CON) or combined (BAC) with a DFM supplement containing a mix of *Bacillus subtilis* 810 and *B. licheniformis* 809 (Bovacillus; Novonesis, Lyngby, Denmark). Soyhulls supplementation was offered to support the BCS gain of all heifers during the prepartum period and to minimize the BCS loss during the postpartum period (NASEM 2016). The commercial blend (Bovacillus; Novonesis, Lyngby, Denmark) was mixed with soybean hulls once a week in quantities designed to provide on average 3 g of the DFM mixture per heifer daily, aiming to achieve an expected total colony forming unit (CFU) of 6.6 × 10^9^ per heifer daily (Cordeiro et al. 2024; Terré et al. 2024; Dias et al. 2022). Treatments were provided from day 0 to 242 of the study (139 ± 4 d prepartum until 104 ± 4 d postpartum). Each supplement (CON and BAC) was hand-delivered daily to the respective pastures at 0800 h in plastic feed bunks positioned 1 m above ground level to prevent calves from consuming the maternal supplements. Heifers that did not give birth to a live calf (*n *= 3 CON and 3 BAC) and heifer-calf pairs that were not early weaned due to being younger than 52 d at the time of weaning (*n *= 4 CON and 2 BAC) were removed from all statistical analyses.

#### Offspring management

On day 242, calves (*n *= 60; 15 heifers and 15 steers per treatment) were early weaned (96 ± 30 d of age), combined in a single group and transferred to a partially covered drylot pen for 16 d to overcome the stress of weaning. Early weaning was adopted to improve the reproductive performance of primiparous *Bos indicus*-influenced beef cows (Arthington and Kalmbacher 2003), while also ensuring comparable post-weaning DM intake among calves and avoiding potential confounding between treatments during the post-weaning phase. On day 258, calves were assigned to 1 of 12 pens (15 × 5 m) within a partially covered drylot facility, following the same previous pasture distribution assigned to their dams (4 to 6 calves per pen). Calves were gradually adapted to concentrate (21% crude protein and 72.5% total digestible nutrients) and then limit-fed the same concentrate at 3.25% of BW (DM basis) until day 319. Concentrate was provided daily at 0800 h. In addition, calves received limpograss (*Hemarthria altissima*) hay at 0.50% of BW (DM basis) with a complete trace mineral supplement from days 258 to 319. The total diet offered during the drylot period was selected based on previous studies (Izquierdo et al. 2023; Moriel et al. 2020) and formulated to support an average daily gain (ADG) of 1 kg/d (NASEM 2016). On day 258, each calf was vaccinated against pathogens associated with bovine respiratory disease (2 mL subcutaneous; Bovi Shield Gold One Shot; Zoetis Inc., New York, NY) and *Clostridium spp.* (2 mL subcutaneous; Ultrabac 7, Zoetis Inc., New York, NY), and received an oral drench of fenbendazole (5 mg/kg of BW; Safe-guard, Merck Animal Health, Summit, NJ). On day 272, calves received booster vaccinations of Bovi Shield Gold 5 and Ultrabac 8 (2 mL subcutaneous; Zoetis Inc.). This vaccination protocol was chosen as a model to elicit an inflammatory response (Izquierdo et al. 2023; Moriel et al. 2020). Calf health was monitored daily by trained personnel from birth until the end of the study.

### Data and sample collection

Individual fecal grab samples (∼15 g; as-fed basis) were collected from 3 heifers per pasture (randomly selected on day 0) on days 0, 90, and 180, corresponding to the start of the study, prepartum period, and postpartum period, respectively. Individual fecal grab samples (∼15 g; as-fed basis) were collected from the respective offspring of the heifers selected for maternal fecal sampling (9 steers and 9 heifers per treatment) on days 242 and 272, corresponding to early weaning and 14 d after drylot entry, during which all calves consumed the same post-weaning diet to minimize confounding effects of dry matter intake and diet composition. Fecal samples of heifers and their calves were obtained directly from the rectum of each animal while restrained in a chute, using a new sterile rectal palpation sleeve for each collection. Samples were immediately transferred to sterile 15-mL tubes, placed on ice, and subsequently stored at −80°C until laboratory analysis.

### Laboratory analyses

#### DNA extraction, purification and quantification

Fecal samples were sent to a commercial laboratory (FERA Diagnostics and Biologicals Corp.; College Station, TX) for DNA extraction and 16S rRNA gene amplicon sequencing. Samples were transferred to a 96-well plate and DNA extraction was performed using Mag-Bind Universal Pathogen 96 Kit (Omega Bio-Tek, Norcross, GA). The 16S amplicons were amplified by polymerase chain reaction (PCR) for individual metagenomic DNA samples as previously described by Bicalho et al. (2017). The bacterial 16S rDNA V4 region was performed using 515F (5ʹ-GTGCCAGCMGCCGCGGTAA-3′ʹ) and 806R (5ʹ-GGACTACHVGGGTWTCTAAT-3ʹ) primers following protocols adapted for Illumina MiSeq platform (Caporaso et al. 2012). Amplicon presence and size were verified by electrophoresis on 1.2% agarose gels stained with 0.5 mg/mL ethidium bromide. PCR products were purified using Mag-Bind^®^ TotalPure NGS (Omega Bio-Tek, Norcross, GA). DNA concentration was determined by spectrophotometry considering that an A260 of 1.0 corresponds to 50 µg/mL of double-stranded DNA.

#### DNA normalization and pooling

Extracted DNA was diluted to uniforms concentrations using ultrapure distilled water. Equal volumes from each sample were pooled using a high-precision liquid-handling robot. The pooled library was quantified uding Qubit^®^ fluorometric quantification (Thermo Fisher Scientific, Waltham, MA) for accurate measurement of double-stranded DNA.

#### Library preparation and sequencing

The pooled library was diluted to 4 nM and denatured according to the MiSeq System Denature and Dilute Libraries Guide (Illumina, San Diego, CA). The denatured library was combined with PhiX Control v3 (Illumina) and sequenced using the 16S Metagenomics Workflow on the Illumina MiSeq platform.

#### Data processing and taxonomic classification

FASTQ files and sequencing reports were generated for each sample. Sequence data were processed using the MiSeq Metagenomics Workflow with the Greengenes database (http://greengenes.lbl.gov/), generating operational taxonomic unit (OTU) tables with relative abundances classified at multiple taxonomic levels (kingdom to species). For the purposes of this study, the phylum, genus and species levels were used. For ease of visualization, any phylum and genus with a mean relative abundance below 1% were grouped into an “Others” category, except for *Bacillus*. Although *Bacillus* accounted for less than 1% of the total abundance, it was reported separately to specifically assess the impact of maternal BAC supplementation on the maternal and offspring fecal microbiome.

#### Bacterial diversity

To unravel the fecal microbiome diversity of the heifers, alpha- and beta-diversity analyses were performed based on the bacterial species level. Alpha diversity measures the diversity within a single sample indicating how many bacterial species are present and how evenly they are distributed and were measured using Shannon and Simpson indexes (Weinroth et al. 2022). Beta-diversity measures the difference in species composition between samples indicating how distinct the microbial communities are and was measured using Bray-Curtis dissimilarity analysis (Weinroth et al. 2022).

### Statistical analysis

Six reprocessed samples (2 CON cows, 1 CON calf, and 3 BAC calves) were removed from the statistical analyses due to having no reads or having too low reads resulting in being classified as an outlier. The OTU data obtained from bioinformatics analysis was used to describe the relative abundances of microbial phylum, genus and species in fecal samples. Each value indicated the percentage relative frequency of reads with rRNA genes annotated to the indicated taxonomic level.

#### Bacterial relative abundance

Pasture or pen were the selected experimental unit. Pasture(maternal treatment) and heifer(pasture) or calf(pen) were considered the random effects. Data were analyzed as a completely randomized design using the MIXED procedure of SAS (SAS Institute Inc., Cary, NC, USA, version 9.4). Phylum and genus relative abundance data were analyzed as repeated measures and tested for fixed effects of maternal treatment, day, and maternal treatment × day. The covariance structure was chosen using the lowest Akaike information criterion, and heifer(pasture) or calf(pen) were included as subjects. Phylum and genus relative abundance data obtained on day 0, calf age, and calf sex were initially included as covariates in all respective statistical analyses. Phylum and genus relative abundance data on day 0 remained in the model as covariate (*P *≤ 0.03) for all heifer phyla and genera data. All results are reported as least-square means. Data were separated using PDIFF if a significant F-test was detected. Significance was set at *P *≤ 0.05 and tendencies were noted if *P *> 0.05 and ≤0.10.

#### Bacterial diversity

All statistical analyses were performed in RStudio version 2025.05.0 (RStudio: Integrated Development Environment for R. Posit Software, PBC, Boston, MA). Alpha diversity indexes (Shannon 1948; Simpson 1949) were calculated using the diversity function from the vegan package based on relative abundance data at the species level. Data were analyzed as repeated measures and tested for fixed effects of maternal treatment, day, and maternal treatment × day interaction, using heifer(pasture) or calf(pen) as random effects. Linear mixed-effects models were used and fitted with the lme function from the nlme package. Type III analysis of variance (ANOVA) was applied with the anova function from the lmerTest package. All results are reported as least-square means and data were separated using pairwise comparisons with the emmeans function. Beta diversity was assessed using Bray-Curtis dissimilarity, calculated with vegdist function from vegan package, based on relative abundance data at the species level. Principal Coordinates Analysis (PCoA) was performed using the cmdscale function. The first two principal coordinates (PCoA1 and PCoA2) were extracted and used to create scatter plots colored by treatment, using the ggplot2 package. The Permutational Multivariate Analysis of Variance (PERMANOVA) was applied using the adonis2 function (vegan package) with 999 permutations. Permutations were restricted by pasture using the how function (permute package). The model included the fixed effects of maternal treatment, day, and the maternal treatment × day interaction. In the ordination plot, 95% confidence ellipses were added using stat_ellipse and the plot was faceted by day of the study. Significance was set at *P *≤ 0.05, and tendencies were noted if *P *> 0.05 and ≤0.10.

## Results

### Maternal microbiome

#### Bacteria phyla relative abundance

The relative abundance of bacteria derived from each individual fecal sample was assigned to 27 different phyla. Effects of maternal treatment × day tended to be detected (*P *= 0.09) for Firmicutes phylum (Table 1). Firmicutes relative abundance on days 0 and 180 did not differ (*P *≥ 0.55) between maternal treatments. Nonetheless, Firmicutes relative abundance on day 90 was lower (*P *< 0.01) for BAC vs. CON heifers. Effects of maternal treatment tended to be detected (*P *= 0.10) for Bacteroidetes phylum (Table 1), as its relative abundance tended to be greater for BAC vs. CON heifers. Effects of day of the study were detected (*P *≤ 0.01) for all phyla relative abundance, except for Others (*P *= 0.32; Table 1). Bacteroidetes and Proteobacteria relative abundance increased from day 0 until 180, while Firmicutes and Cyanobacteria decreased from day 0 until 180. Euryarchaeota relative abundance increased from day 0 to 90 and decreased from day 90 to 180.

**Table 1.** Bacterial phyla composition (relative abundance, %) in the fecal samples of heifers offered soybean hulls supplementation (1 kg/d) added (BAC) or not (CON) with a DFM supplement (3 grams per day) containing a combination of *Bacillus subtilis* and *B. licheniformis* (6.6 × 10^9^ CFU per day) from days 0 to 242 (six pastures per treatment).^a^

	Maternal treatment				*P*-value	
Item^b^	CON	BAC	SEM	*P* ^c^	Treatment	Treatment × day
**Firmicutes**						
** Day 0**	69.8	71.1	3.16	0.68	-	0.09
** Day 90**	65.6	57.1	3.16	<0.01	-	
** Day 180 **	50.8	48.9	3.29	0.55	-	
**Bacteroidetes**	27.2	30.2	1.25	-	0.10	0.17
**Proteobacteria**	3.51	3.66	0.105	-	0.33	0.37
**Euryarchaeota**	2.77	2.52	0.317	-	0.57	0.75
**Cyanobacteria**	1.24	1.17	0.078	-	0.53	0.40
**Others**	3.13	3.52	0.203	-	0.20	0.23

a Heifers were sampled for fecal microbiota analysis on days 0 (prior to start of CON and BAC supplementation), 90 and 180. Maternal treatments were provided on average for 139 ± 4 d prepartum and 104 ± 4 d postpartum (days 0 to 242). Heifers were provided free-choice access to limpograss hay and 12.7 kg per week of sugarcane molasses and urea from days 242 to 312. Calves were weaned on day 242 at 96 ± 30 d of age. After weaning, heifers were combined into a single group and placed with three Brangus bulls from days 242 to 312.

b Means covariate-adjusted (*P *≤ 0.02) for the respective baseline value obtained on day 0.

c *P*-value for the comparison of maternal treatment within day of the study.

#### Bacteria genera relative abundance

The relative abundance of bacteria in each individual fecal sample was assigned to 643 different genera. Effects of maternal treatment × day were detected (*P *= 0.05) for *Clostridium* and tended (*P *= 0.09) to be detected for *Blautia* genera (Table 2). Relative abundances of *Clostridium* and *Blautia* on days 0 and 180 did not differ (*P *≥ 0.43) between maternal treatments. *Clostridium* and *Blautia* relative abundances on day 90 were lower (*P *≤ 0.01) for BAC vs. CON heifers. Within the *Clostridium* genera, *Clostridium butyricum* and *C. perfringens* relative abundances did not differ (*P *≥ 0.30) between maternal treatments. Effects of maternal treatment were detected (*P *= 0.03) for *Mogibacterium* and tended to be detected (*P *≤ 0.08) for *Bacteroides* and *Porphyromonas* genera (Table 2). Average *Mogibacterium* relative abundance was lower (*P *= 0.03) for BAC vs. CON heifers, whereas *Bacteroides* and *Porphyromonas* relative abundances tended (*P *≤ 0.08) to be greater for BAC vs. CON heifers. Effects of day of the study were detected (*P *< 0.01) for 14 genera and tended to be detected (*P *≤ 0.09) for *Oscillospira* and Others (Table 2). *Bacteroides*, *Pedobacter*, *Porphyromonas, Paraprevotella*, *Prevotella*, and *Clostridium perfringens* relative abundances increased (*P *< 0.01) from day 0 to 180, whereas *Blautia*, *Caloramator*, *Slackia, Butyrivibrio, Mogibacterium*, *Methanobrevibacter* and *Alkaliphilus* decreased (*P *< 0.01) from day 0 to 180. *Rhodothermus* relative abundance decreased (*P *< 0.01) from day 0 to 90 and increased (*P *< 0.01) from day 90 to 180, whereas *Clostridium butyricum* relative abundance increased (*P *< 0.01) from day 0 to 90 and decreased (*P *= 0.01) from day 90 to 180. *Bacillus* relative abundance decreased (*P *= 0.01) from day 0 to 90. *Oscillospira* relative abundance increased (*P *= 0.04) from day 90 to 180, whereas Others decreased (*P *= 0.03) from day 0 to 180 and tended to be decreased (*P *= 0.09) from day 90 to 180.

**Table 2.** Bacterial genera composition (relative abundance, %) in the fecal samples of heifers offered soybean hulls supplementation (1 kg/d) added (BAC) or not (CON) with a DFM supplement (3 grams per day) containing a combination of *Bacillus subtilis* and *B. licheniformis* (6.6 × 10^9^ CFU per day) from days 0 to 242 (six pastures per treatment).^a^

	Maternal treatment				*P*-value	
Item^b^	CON	BAC	SEM	*P* ^c^	Treatment	Treatment × day
***Bacteroides***	12.1	13.7	0.586	-	0.06	0.28
***Ruminococcus***	13.2	12.7	0.275	-	0.17	0.79
***Blautia***						
** Day 0**	5.26	5.44	0.380	0.64	-	0.09
** Day 90**	5.91	4.97	0.380	0.01	-	
** Day 180**	4.32	4.20	0.398	0.76	-	
***Clostridium***						
** Day 0**	7.95	8.01	0.198	0.87	-	0.05
** Day 90**	7.45	6.18	0.198	<0.01	-	
** Day 180**	6.51	6.19	0.207	0.43	-	
***Pedobacter***	6.33	7.01	0.397	-	0.25	0.19
***Caloramator***	5.32	4.90	0.257	-	0.27	0.35
***Slackia***	5.97	5.47	0.407	-	0.39	0.52
***Butyrivibrio***	2.65	2.35	0.133	-	0.11	0.68
***Oscillospira***	3.20	3.09	0.073	-	0.29	0.91
***Mogibacterium***	3.83	3.40	0.143	-	0.03	0.19
***Methanobrevibacter***	2.97	2.72	0.337	-	0.60	0.76
***Porphyromonas***	1.84	2.14	0.112	-	0.08	0.26
***Paraprevotella***	1.47	1.58	0.132	-	0.54	0.44
***Rhodothermus***	1.53	1.56	0.047	-	0.70	0.27
***Prevotella***	1.38	1.33	0.109	-	0.74	0.26
***Alkaliphilus***	1.08	1.05	0.028	-	0.42	0.70
***Natronincola***	1.14	1.30	0.142	-	0.42	0.77
***Bacillus***	0.122	0.137	0.011	-	0.39	0.92
**Others**	23.3	24.0	0.382	-	0.24	0.65

a Heifers were sampled for fecal microbiota analysis on days 0 (prior to start of CON and BAC supplementation), 90 and 180. Maternal treatments were provided on average for 139 ± 4 d prepartum and 104 ± 4 d postpartum (days 0 to 242). Heifers were provided free-choice access to limpograss hay and 12.7 kg per week of sugarcane molasses and urea from days 242 to 312. Calves were weaned on day 242 at 96 ± 30 d of age. After weaning, heifers were combined into a single group and placed with three Brangus bulls from days 242 to 312.

b Means covariate-adjusted (*P *≤ 0.03) for the respective baseline value obtained on day 0.

c *P*-value for the comparison of maternal treatment within day of the study.

#### Bacteria species diversity

**Fig. 1.**
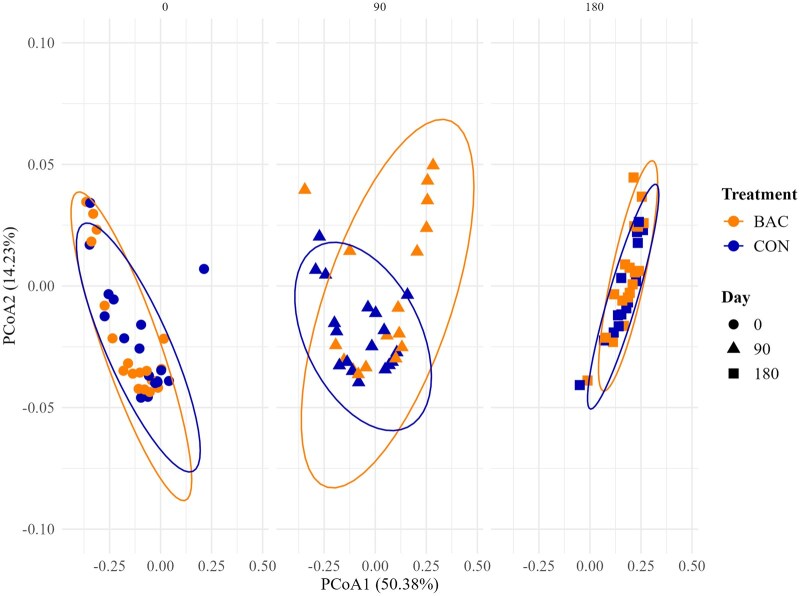
Principal coordinates analysis (PCoA) based on bray–curtis distances across time (days 0, 90 and 180) in the fecal samples of heifers offered soybean hulls supplementation (1 kg/d) added (BAC) or not (CON) a DFM supplement (3 g per day) containing a combination of *Bacillus subtilis* and *B. licheniformis* (6.6 × 10^9^ CFU per day) from days 0 to 242 (six pastures per treatment). The contribution rates of PCoA1 and PCoA2 were 50.4% and 14.2%, respectively.

The relative abundance of bacteria species derived from each individual fecal sample was assigned to 1404 different species. Effects of day and maternal treatment × day (*P *≥ 0.20) were not detected for both Shannon and Simpson diversity indexes (Table 3). Effects of maternal treatment tended to be detected (*P *= 0.09) for Shannon and Simpson diversity indexes (Table 3) and were greater for BAC vs. CON heifers. Overall, both indexes remained stable across time, with Shannon values ranging from approximately 3.60 to 3.85 and Simpson values around 0.944, indicating consistent microbial richness and evenness (Table 3). Bray-Curtis analysis showed no clear segregation between maternal treatments across time on the Principal Coordinates Analysis (PCoA), although distinct temporal patterns were observed (Fig. 1). PERMANOVA detected effects of maternal treatment (*P *≤ 0.01, R^2^ = 0.007) and day (*P *< 0.01, R^2^ = 0.254) on microbial composition. Effects of maternal treatment × day were not detected (*P *= 0.18, R^2^ = 0.020) for bacterial beta-diversity.

**Table 3.** Alpha-diversity indexes across time in the fecal samples of heifers offered soybean hulls supplementation (1 kg/d) added (BAC) or not (CON) with a DFM supplement (3 g per day) containing a combination of *Bacillus subtilis* and *B. licheniformis* (6.6 × 10^9^ CFU per day) from days 0 to 242 (six pastures per treatment).^a^

	Maternal treatment			*P*-value	Day of the study				*P*-value
Itemb	CON	BAC	SEM	Treatment	0	90	180	SEM	Day
**Shannon Index**	3.74	3.77	0.094	0.09	3.71	3.81	3.75	0.099	0.78
**Simpson Index**	0.936	0.952	0.022	0.09	0.927	0.955	0.952	0.022	0.99

a Heifers were sampled for fecal microbiota analysis on days 0 (prior to start of CON and BAC supplementation), 90 and 180. Maternal treatments were provided on average for 139 ± 4 d prepartum and 104 ± 4 d postpartum (days 0 to 242). Heifers were provided free-choice access to limpograss hay and 12.7 kg per week of sugarcane molasses and urea from days 242 to 312. Calves were weaned on day 242 at 96 ± 30 d of age. After weaning, heifers were combined into a single group and placed with three Brangus bulls from days 242 to 312.

### Offspring microbiome

#### Bacteria phyla relative abundance

The relative abundance of bacteria derived from each individual fecal sample was assigned to 29 different phyla. Effects of maternal treatment × day tended (*P *= 0.08) to be detected for Proteobacteria phylum (Table 4). Proteobacteria relative abundance on day 242 tended (*P *= 0.09) to be greater for BAC vs. CON calves and did not differ (*P *= 0.68) between maternal treatments on day 272. Within the Proteobacteria phylum, *Salmonella enterica* and *Escherichia coli* relative abundances did not differ (*P *≥ 0.28) between maternal treatments. Effects of maternal treatment were detected (*P *≤ 0.01) for Firmicutes and Bacteroidetes and tended (*P *= 0.10) to be detected for Euryarchaeota phyla (Table 4). Firmicutes relative abundance was lower (*P *< 0.01) for BAC vs. CON calves, while Bacteroidetes was greater (*P *= 0.01) for BAC vs. CON calves. Euryarchaeota relative abundance tended (*P *= 0.10) to be lower for BAC vs. CON calves. Effects of day were detected (*P *≤ 0.02) for all phyla relative abundance, except for Firmicutes (*P *= 0.13; Table 4). Euryarchaeota and Others relative abundance increased (*P *< 0.01) from day 242 to 272, while Bacteroidetes, Proteobacteria, and *E. coli* decreased (*P *≤ 0.02) from day 242 to 272. *Salmonella enterica* relative abundance tended to increase (*P *= 0.07) from day 242 to 272.

**Table 4.** Bacterial phyla composition (relative abundance, %) in the fecal samples of offspring born from heifers offered soybean hulls supplementation (1 kg/d) added (BAC) or not (CON) with a DFM supplement (3 grams per day) containing a combination of *Bacillus subtilis* and *B. licheniformis* (6.6 × 10^9^ CFU per day) from days 0 to 242 (six pastures per treatment).^a^

Item^b^	Maternal treatment				*P*-value	
	CON	BAC	SEM	*P* ^c^	Treatment	Treatment × day
**Firmicutes**	65.2	57.4	2.00	-	< 0.01	0.81
**Bacteroidetes**	26.4	33.4	1.96	-	0.01	0.80
**Proteobacteria**						
** Day 242**	3.47	6.36	1.69	0.09	-	0.08
** Day 272**	2.61	2.48	0.315	0.68	-	
**Euryarchaeota**	2.09	1.59	0.214	-	0.10	0.79
**Others**	3.32	3.27	0.347	-	0.92	0.85

a Calves were sampled for fecal microbiota analysis on days 242 (weaning) and 272. Maternal treatments were provided on average for 139 ± 4 d prepartum and 104 ± 4 d postpartum (days 0 to 242). Calves were weaned on day 242 at 96 ± 30 d of age. From days 242 to 257, calves remained in a single drylot pen and were offered free-choice access to limpograss hay and 0.50 kg/d of a pelletized supplement. On day 258, calves were transferred to 1 of 12 drylot pens using the previous maternal pasture assignment distribution (4 to 6 calves/pen). Starting on day 258, calves were gradually adapted to concentrate by increasing diet DM offered by 0.25% to 0.50% of BW/d for 7 d. Then, calves were limit-fed the same concentrate at 3.25% of BW and limpograss hay at 0.5% of BW (DM basis) until day 319.

b Calf age and sex were removed from the model of all statistical analyses of calf variables (*P *≥ 0.29).

c *P*-value for the comparison of maternal treatment within day of the study.

#### Bacteria genera relative abundance

The relative abundance of bacteria derived from each individual fecal sample was assigned to 656 different genera. Effects of maternal treatment × day tended (*P *= 0.06) to be detected for *Paraprevotella* genus (Table 5). *Paraprevotella* relative abundance on day 242 was lower (*P *= 0.05) for BAC vs. CON calves, and did not differ (*P *= 0.89) between maternal treatments on day 272. Effects of maternal treatment were detected (*P *≤ 0.01) for *Bacteroides* and *Slackia* genera and tended to be detected (*P *≤ 0.08) for *Blautia, Butyrivibrio*, and *Methanobrevibacter* genera (Table 5). *Bacteroides* relative abundance was greater (*P *= 0.01) for BAC vs. CON calves, while *Slackia* was lower (*P *< 0.01) for BAC vs. CON calves. *Blautia, Butyrivibrio*, and *Methanobrevibacter* relative abundance tended (*P *= 0.08) to be lower for BAC vs. CON calves. Within *Clostridium* genera, *Clostridium butyricum* and *C. perfringens* relative abundances did not differ (*P *≥ 0.21) between maternal treatments. Effects of day were detected (*P *< 0.03) for 14 genera and tended (*P *≤ 0.07) for *Bacillus* genera and *Clostridium perfringens* (Table 5). *Caloramator, Slackia, Butyrivibrio, Oscillospira, Mogibacterium, Methanobrevibacter, Porphyromonas, Rhodothermus*, and *Alkaliphilus* relative abundances increased (*P *≤ 0.03) from day 242 to 272, while *Bacteroides, Blautia, Prevotella, Faecalibacterium*, and *Lactobacillus* decreased (*P *≤ 0.02) from day 242 to 272. *Bacillus* and *Clostridium perfringens* relative abundances tended to decrease (*P *≤ 0.07) from day 242 to 272.

**Table 5.** Bacterial genera composition (relative abundance, %) in the fecal samples of offspring born from heifers offered soybean hulls supplementation (1 kg/d) added (BAC) or not (CON) with a DFM supplement (3 grams per day) containing a combination of *Bacillus subtilis* and *B. licheniformis* (6.6 × 10^9^ CFU per day) from days 0 to 242 (six pastures per treatment).^a^

	Maternal treatment				*P-*value	
Item^b^	CON	BAC	SEM	*P* ^c^	Treatment	Treatment × day
***Bacteroides***	11.6	17.7	1.58	-	0.01	0.31
***Ruminococcus***	11.0	10.7	0.825	-	0.81	0.86
***Blautia***	11.2	8.98	0.827	-	0.06	0.54
***Clostridium***	6.51	5.73	0.611	-	0.36	0.36
***Pedobacter***	3.53	4.80	0.566	-	0.12	0.65
***Caloramator***	4.47	4.14	0.325	-	0.48	0.43
***Slackia***	1.53	1.01	0.117	-	< 0.01	0.61
***Butyrivibrio***	5.90	4.19	0.693	-	0.08	0.24
***Oscillospira***	2.98	3.05	0.285	-	0.86	0.29
***Mogibacterium***	1.94	1.62	0.146	-	0.11	0.44
***Methanobrevibacter***	1.99	1.46	0.214	-	0.08	0.56
***Porphyromonas***	1.53	1.66	0.233	-	0.68	0.32
***Paraprevotella***						
** Day 242**	4.30	2.18	1.06	0.05	-	0.06
** Day 272**	1.30	1.38	0.597	0.89	-	
***Streptococcus***	3.55	3.59	0.909	-	0.97	0.82
***Rhodothermus***	1.24	1.49	0.152	-	0.26	0.56
***Prevotella***	1.58	1.56	0.401	-	0.97	0.88
***Alkaliphilus***	1.19	1.15	0.139	-	0.80	0.75
***Faecalibacterium***	1.14	1.48	0.523	-	0.64	0.64
***Parabacteroides***	1.83	2.03	0.453	-	0.75	0.96
***Lactobacillus***	1.85	0.980	0.504	-	0.23	0.25
***Bacillus***	0.068	0.074	0.018	-	0.83	0.42
**Others**	20.3	21.0	1.10	-	0.67	0.32

a Calves were sampled for fecal microbiota analysis on days 242 (weaning) and 272. Maternal treatments were provided on average for 139 ± 4 d prepartum and 104 ± 4 d postpartum (days 0 to 242). Calves were weaned on day 242 at 96 ± 30 d of age. From days 242 to 257, calves remained in a single drylot pen and were offered free-choice access to limpograss hay and 0.50 kg/d of a pelletized supplement. On day 258, calves were transferred to 1 of 12 drylot pens using the previous maternal pasture assignment distribution (4 to 6 calves/pen). Starting on day 258, calves were gradually adapted to concentrate by increasing diet DM offered by 0.25% to 0.50% of BW/d for 7 d. Then, calves were limit-fed the same concentrate at 3.25% of BW and limpograss hay at 0.5% of BW (DM basis) until day 319.

b *Faecalibacterium* e *Parabacteroides* relative abundances were covariate-adjusted for calf age (*P *≤ 0.03). Calf age and sex were removed from the model of all remaining statistical analyses of calf variables (*P *≥ 0.17).

c *P*-value for the comparison of maternal treatment within day of the study.

#### Bacteria species diversity

**Fig. 2.**
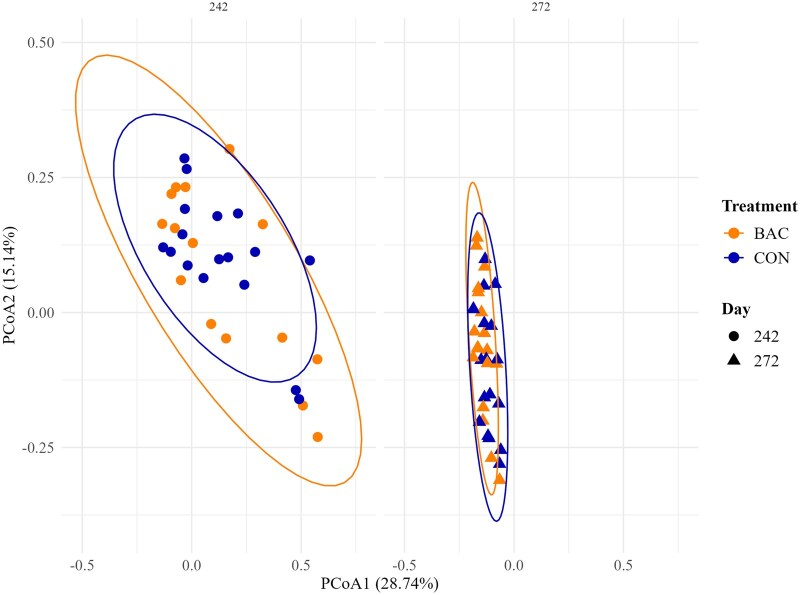
Principal coordinates analysis (PCoA) based on bray–curtis distances across time (days 242 and 272) in the fecal samples of offspring born from heifers offered soybean hulls supplementation (1 kg/d) added (BAC) or not (CON) with a DFM supplement (3 g per day) containing a combination of *Bacillus subtilis* and *B. licheniformis* (6.6 × 10^9^ CFU per day) from days 0 to 242 (six pastures per treatment). Orange points represent the BAC offsprings and blue points represent the CON offsprings. The contribution rates of PCoA1 and PCoA2 were 28.7% and 15.1%, respectively.

The relative abundance of bacteria derived from each individual fecal sample was assigned to 1,486 different species. Effects of maternal treatment × day and maternal treatment were not detected (*P *≥ 0.14), whereas effects of day were detected (*P *< 0.01) for Shannon diversity index (Table 6). Effects of maternal treatment × day were detected (*P *= 0.05) for Simpson diversity index (Table 6), which remained constant (*P *= 0.98) for CON calves from day 242 to 272 but increased (*P *= 0.02) for BAC calves from day 242 to 272. Both indexes remained stable across time, with Shannon values ranging from approximately 3.36 to 3.67 and Simpson values around 0.932, indicating consistent microbial richness and evenness (Table 6). Bray-Curtis analysis showed no clear segregation between maternal treatments across time on the PCoA, although distinct temporal patterns were observed (Fig. 2). PERMANOVA detected effects of maternal treatment (*P *< 0.01, R^2^ = 0.012) and day of the study (*P *< 0.01, R^2^ = 0.178) on microbial composition, but no effects of maternal treatment × day were detected (*P *= 0.36, R^2^ = 0.013).

**Table 6.** Alpha-diversity indexes across time (days 242 and 272) in the fecal samples of offspring born from heifers offered soybean hulls supplementation (1 kg/d) added (**BAC**) or not (**CON**) a DFM supplement (3 g per day) containing a combination of *Bacillus subtilis* and *B. licheniformis* (6.6 × 10^9^ CFU per day) from days 0 to 242 (six pastures per treatment).^a^

	Maternal treatment					*P*-value
Item	CON	BAC	SEM	*P*-value^b^	Treatment × day	Treatment
**Shannon Index**	3.55	3.52	0.081	-	0.14	0.18
**Simpson Index**					0.05	0.05
** Day 242**	0.934	0.908	0.019	0.25	-	-
** Day 272**	0.939	0.949	0.009	0.86	-	-

a Calves were sampled for fecal microbiota analysis on days 242 (weaning) and 272. Maternal treatments were provided on average for 139 ± 4 d prepartum and 104 ± 4 d postpartum (days 0 to 242). Calves were weaned on day 242 at 96 ± 30 d of age. From days 242 to 257, calves remained in a single drylot pen and were offered free-choice access to limpograss hay and 0.50 kg/d of a pelletized supplement. On day 258, calves were transferred to 1 of 12 drylot pens using the previous maternal pasture assignment distribution (4 to 6 calves/pen). Starting on day 258, calves were gradually adapted to concentrate by increasing diet DM offered by 0.25% to 0.50% of BW/d for 7 d. Then, calves were limit-fed the same concentrate at 3.25% of BW and limpograss hay at 0.5% of BW (DM basis) until day 319.

b *P*-value for the comparison of maternal treatment within day of the study.

## Discussion

### Maternal microbiome

Heifers supplemented with *Bacillus*-based DFM demonstrated greater prepartum BCS gain and higher BCS at calving compared to controls (Izquierdo et al. 2024), along with significant alterations in their prepartum metabolome and metabolic pathways (Izquierdo et al. 2025). It is well established that maternal nutrition during gestation triggers a cascade of metabolic adaptations that can influence both maternal and offspring physiology (Moriel et al. 2021). The present study highlights the complexity of the gut microbiota and provides novel evidence that *Bacillus*-based DFM supplementation modulates bacterial communities in a manner that may have contributed to the improved maternal ­prepartum BCS observed in BAC vs. CON heifers (Izquierdo et al. 2024).

No effects of maternal treatment were detected on the relative abundance of *Bacillus* in either heifers or their offspring. This finding aligns with the understanding that supplementation with *Bacillus*-based DFM does not necessarily lead to increased fecal detection of *Bacillus* spp., since the strains used as probiotics are spore-forming and act primarily as transient members of the microbial community rather than as stable gut colonizers (Bernardeau et al. 2017). Despite this*, Bacillus*-based DFM supplementation can still influence the broader microbial population within the gastrointestinal tract, as demonstrated in previous studies (Lamontagne et al. 2023; Linde et al. 2023; Limede et al. 2025).

Consistent with previous studies (Kim et al. 2014; Myer et al. 2015), the predominant bacterial phyla identified in both heifers and their offspring were Firmicutes, Bacteroidetes, and Proteobacteria. The Firmicutes phylum is primarily associated with carbohydrate fermentation and butyrate production in the gastrointestinal tract, processes that are critical for maintaining gut epithelial integrity and supporting host energy metabolism (Singh et al. 2022). Within this phylum, the genera *Blautia* and *Clostridium* were less abundant in BAC heifers compared with CON heifers on day 90. The *Blautia* genus has been recognized for its role in promoting intestinal health (Liu et al. 2021). In contrast, *Clostridium* species encompass both beneficial strains, such as *Clostridium butyricum*, which produces butyrate and supports mucosal health, and potentially pathogenic strains, such as *Clostridium difficile* and *Clostridium perfringens*, which may negatively affect gut health and reproductive performance (Kronfeld et al. 2022). *Bacillus*-based DFM can selectively suppress the negative health effects of pathogenic *Clostridium* species (Guimaraes et al. 2023), while promoting beneficial fermenters through competitive exclusion, antimicrobial metabolite production, and improved substrate availability (Luise et al. 2022). In the current study, *C. difficile* was not detected and no treatment effects were observed for the relative abundances of *C. butyricum* and *C. perfringens* on maternal fecal samples. However, microbial diversity and composition in the rumen and hindgut require time to be modulated by dietary interventions (Weilmet, 2015  Weimer et al. 2015) and can also be influenced by BCS (Saraphol et al. 2025). ­Additionally, environmental factors such as temperature-humidity index (THI) affect microbial communities. For instance, Li et al. (2020) demonstrated that variations in THI modulate gut microbial structure, and Calamari et al. (2018) reported that heat exposure altered fecal fermentative parameters and hindgut buffering capacity, increasing the occurrence of *Clostridium tyrobutyricum* spores in feces. In the present study, BAC heifers exhibited a lower relative abundance of *Clostridium* on day 90 but not on day 180 compared with CON heifers. These temporal responses can be explained by both biological and environmental factors. From day 90 to 180, both treatments experienced a loss in BCS (Izquierdo et al. 2024), which may have influenced the bacterial community. Moreover, day 90 corresponded to the summer period (average THI = 79; Izquierdo et al. 2024), when warm and humid ­conditions favor *Clostridium* growth, whereas day 180 corresponded to fall (average THI = 73; Izquierdo et al. 2024), ­characterized by cooler and less humid conditions. These seasonal changes may have masked the DFM-induced reduction of *Clostridium* populations in feces on day 180.

The observed reduction in *Mogibacterium* (Firmicutes phylum) in BAC heifers, though not fully understood, is consistent with previous findings (Berding et al. 2016). On day 90, the decrease in Firmicutes abundance was accompanied by a tendency toward greater average relative abundance of Bacteroidetes, including the genera *Bacteroides* and *Porphyromonas*, in BAC compared with CON heifers. Bacteroidetes play a key role in degrading starch, fiber, and dietary proteins, thereby facilitating intestinal absorption of amino acids and peptides (Thomas et al. 2011). This phylum also produces short-chain fatty acids (SCFA), such as acetate and propionate, which are central to host energy metabolism and immune regulation (Flint et al. 2012). The tendency for higher Bacteroidetes abundance in BAC heifers therefore suggests an enhanced hindgut fermentative capacity, potentially improving metabolic efficiency and intestinal function (Cappellozza et al. 2023). Collectively, these shifts imply that *Bacillus*-based DFM supplementation modulates the gastrointestinal microbiota by reducing potentially dysbiotic Firmicutes populations while promoting beneficial Bacteroidetes. Dysbiosis is typically characterized by reduced microbial diversity in the rumen (Petri et al. 2013) and a ­disproportionately high abundance of Proteobacteria (Auffret et al. 2017). Probiotics are known to exert stabilizing effects on rumen microbiome composition (Lamontagne et al. 2023; Linde et al. 2023). Because dysbiosis compromises microbial community stability, pathogenic bacteria can proliferate, negatively affecting host health. Supplementation with probiotics has been shown to improve microbial diversity, richness, and abundance, which in turn enhances immunity, reduces the incidence of metabolic disorders, and promotes nutrient digestion and absorption (Du et al. 2018). Such microbial remodeling likely alters SCFA profiles, specifically by increasing acetate production while potentially reducing butyrate concentrations, which may have contributed to the improved prepartum BCS gain observed in BAC heifers (Izquierdo et al. 2024). The relationship between gut microbiota and SCFA concentrations highlights the link between probiotic supplementation and fermentation profiles (Besten et al. 2013). Acetate, the primary SCFA produced through ruminal fermentation of fiber carbohydrates, is the main precursor for de novo lipogenesis in ruminants. Once absorbed, acetate is utilized by adipose tissue for fat synthesis, directly contributing to energy storage and improved BCS (Piantoni and VandeHaar 2022). In contrast, butyrate is largely metabolized to ketone bodies in the rumen epithelium, playing a more limited role in systemic fat deposition (Baldwin and Allison 1983). Therefore, a fermentation shift favoring acetate over butyrate reflects a more lipogenic profile, supporting greater energy storage. These findings align with the reported benefits of combining *Bacillus licheniformis* 809 and *Bacillus subtilis* 810 on fermentation dynamics and nutrient utilization efficiency (Cappellozza et al. 2025; Limede et al. 2025; Pan et al. 2022). Furthermore, butyrate can influence the growth of *Clostridioides difficile*, a member of the *Clostridium* genus, by acting as a metabolic signal that modulates toxin production and sporulation (Pensinger et al. 2023). Thus, shifts in SCFA composition (particularly reduced butyrate) may ­contribute indirectly to limiting the proliferation of *Clostridium* spp. in the gastrointestinal tract as an effect of DFM supplementation.

Analysis of bacterial alpha-diversity (ie, Shannon and Simpson indexes) revealed that BAC heifers tended to exhibit greater microbial richness and evenness than CON heifers during the late prepartum and early postpartum periods. Since dysbiosis is typically characterized by reduced diversity (Petri et al. 2013), these trends suggest that maternal BAC supplementation supported a more diverse and balanced gut microbiota in heifers. Such diversity is particularly critical during gestation, as it contributes to intestinal stability, nutrient metabolism, immune function, and pathogen resistance (Arthur et al. 2025; Campbell et al. 2025; Pickard et al. 2017). The relative stability of both diversity indices over time further indicates that the microbiome remained consistent and that the modulatory effects of BAC supplementation may have persisted throughout the supplementation period. Beta-diversity analyses provided additional evidence for treatment effects on the maternal microbiota. The PERMANOVA analyses revealed significant influences of both maternal treatment and sampling day on microbial community composition, though no interaction was detected. Interestingly, BAC heifers exhibited greater beta-diversity dispersion compared with CON heifers, suggesting that *Bacillus*-based DFM supplementation promoted more individualized microbial profiles, likely reflecting host-specific responses to supplementation. Furthermore, the temporal pattern of beta-diversity (characterized by greater dispersion from day 0 to 90, followed by reduced dispersion from day 90 to 180) indicates dynamic microbial shifts. This pattern may reflect adaptation of the microbiota to late gestation and postpartum physiological changes, followed by stabilization during the later postpartum period (Liu et al. 2024; Lima et al. 2015; Zhu et al. 2017).

### Offspring microbiome

The neonatal gastrointestinal tract is sterile in utero but rapidly colonized during and after birth, which is a critical period for gut and immune system development influenced by early exposure to maternal microbiota and diet (Fanaro et al. 2003; Fouhy et al. 2012; Hansen et al. 2012; Rodríguez et al. 2015). Maternal supplementation with *Bacillus*-based DFM during gestation and lactation enhanced calf average daily gain, feed efficiency, and humoral immune response to vaccination against pathogens associated with bovine respiratory disease during the post-weaning drylot phase (Izquierdo et al. 2024). Notably, calves were not provided direct access to maternal DFM supplementation from birth through early weaning and did not receive DFM supplementation during the post-weaning drylot period. Moreover, calves were limit-fed the same diet starting 14 d before the first vaccination, removing diet DM intake as a potential confounding factor (Izquierdo et al. 2024). The improved post-weaning performance of BAC offspring, alongside changes in their plasma metabolome (Izquierdo et al. 2025), may partly reflect the greater prepartum BCS gain of BAC vs. CON heifers (Izquierdo et al. 2024). Additionally, maternal-offspring microbial interactions occurring from birth through weaning are also known to influence offspring development (Chu et al. 2016). Thus, we hypothesized that such improvements in calf performance could also partially be influenced by the carryover effects of maternal BAC supplementation on offspring fecal microbiome. As described below, calves born to BAC heifers exhibited microbiota shifts similar to those observed in their dams, despite the absence of direct DFM consumption. These findings suggest the occurrence of vertical or early-life microbial transfer from dam to offspring. Such transfer may have taken place through direct or indirect ingestion of maternal fecal microbiota, for instance, via fecal contamination of the pasture consumed by calves or through licking behavior by the dams, given the similarity between salivary and ruminal microbiota (Zhuang et al. 2024). Early-life exposure to specific microbial communities is known to promote long-term colonization (Yáñez-Ruiz et al. 2015), which could explain the persistence of most microbial shifts throughout the calf evaluation period, despite similar dietary intake and the absence of direct DFM supplementation. These enduring microbial changes likely contributed to the improved growth performance and enhanced post-vaccination humoral immune responses observed in BAC compared with CON calves (Izquierdo et al. 2024). Collectively, these results highlight the potential of *Bacillus*-based DFM supplementation in dams to modulate the microbial ecosystems of heifer–calf pairs, even when only the maternal diet is supplemented.

Similar to their dams, BAC calves exhibited a lower average relative abundance of Firmicutes and a higher abundance of Bacteroidetes, particularly the genus *Bacteroides*, compared with CON calves. The enrichment of *Bacteroides* in BAC calves likely supported SCFA production, especially propionate, while also enhancing intestinal barrier function and integrity and contributing to the regulation of innate immune responses (Kiernan et al. 2023; Peng et al. 2009). Within Firmicutes, butyrate-producing genera such as *Blautia* and *Butyrivibrio* were less abundant in BAC vs. CON calves. Approximately 90% of ruminal butyrate is metabolized by the rumen epithelium into ketone bodies and CO_2_ (Bergman 1990), and excess butyrate can inhibit hepatic utilization of propionate, thereby impairing gluconeogenesis and reducing glucose availability (Aiello et al. 1989). Thus, a reduction in butyrate-producing bacteria may indicate a metabolic shift toward greater propionate availability and improved energy efficiency. In addition to Firmicutes, BAC calves tended to have a reduced abundance of Euryarchaeota, primarily *Methanobrevibacter*, compared with CON calves. *Methanobrevibacter* plays a central role in methane production by utilizing hydrogen generated during carbohydrate fermentation, thereby influencing both host energy efficiency and enteric methane emissions (McGovern et al. 2017). Lower *Methanobrevibacter* abundance in BAC calves may therefore reflect shifts in fermentation pathways with potential energetic benefits. Other taxa were also reduced in BAC calves. The genus *Slackia* (Actinobacteria), although less abundant and functionally relevant in ruminants (Nagai et al. 2010), participates in bile acid and bioactive compound metabolism in other hosts. Its reduction may reflect changes in substrate availability or altered microbial interactions (Nagai et al. 2010). Similarly, *Paraprevotella* (Bacteroidetes), despite its role in carbohydrate fermentation, has been associated with microbial dysbiosis and inflammatory responses in ruminants (Mao et al. 2015). Its reduced abundance may therefore indicate a more stable intestinal environment and could have contributed to the improved post-vaccination humoral immune response observed in BAC vs. CON calves (Izquierdo et al. 2024). Taken together, the microbial shifts in calf fecal samples suggest a subtle DFM-induced transition from butyrate- to propionate-producing bacteria, enhancing host energy metabolism and supporting calf performance. Alongside the reduced abundance of microorganisms associated with inflammation and inefficient fermentation, these carryover effects of maternal BAC supplementation on offspring microbiota may underlie the observed improvements in post-weaning growth performance, humoral immunity (Izquierdo et al. 2024), and plasma metabolome (Izquierdo et al. 2025) compared with CON calves.

Conversely, the relative abundance of Proteobacteria was unexpectedly higher in BAC calves at the time of early weaning (day 242) but did not differ on day 272 after calves were consuming the same diet. Proteobacteria include typical intestinal pathogens such as *Escherichia coli* and *Salmonella enterica*, making this increase somewhat surprising. *Bacillus* spp. effectively reduced the abundance of *Salmonella* and *Escherichia coli* under *in vitro* conditions (Capern et al. 2025; Copani et al. 2024). In the present study, no treatment-induced differences were observed for the relative abundances of *Salmonella enterica* and *Escherichia coli* between CON and BAC offspring. Consistent with the findings in heifers, the relative abundances of *C. butyricum* and *C. perfringens* did not differ between CON and BAC calves, likely due to early-life microbial transfer from dam to offspring.

No significant effects of maternal treatment or treatment × day were observed for the Shannon diversity index in calves, indicating that overall bacterial richness and diversity remained similar between groups. However, a significant interaction was detected for the Simpson index, driven by a marked increase in species evenness over time in BAC calves. This suggests that the gut microbiota of BAC calves became more compositionally balanced, with fewer dominant species and a more equitable distribution of taxa. Early-life microbial diversity is critical for immune, digestive, and metabolic development (Hansen et al. 2012), and greater species evenness is often associated with a more stable and resilient microbial ecosystem (Werner et al. 2014). Beta-diversity analyses revealed that both maternal treatment and sampling day influenced the fecal microbial composition of calves. The greater dispersion in beta-diversity observed in BAC calves indicates higher inter-individual variability in microbial community structure, suggesting that maternal BAC supplementation elicited more individualized microbial responses in the offspring, potentially reflecting stronger host-specific modulation of the microbiota. From day 242 to 272, beta-diversity dispersion decreased across all calves, indicating a temporal convergence and stabilization of the gut microbiota, likely driven by a uniform post-weaning diet and shared environmental conditions.

In conclusion, maternal supplementation with a *Bacillus*-based DFM during gestation and early lactation effectively modulated the fecal microbiota of both heifers and their offspring. In heifers, supplementation promoted shifts in microbial composition characterized by increased Bacteroidetes abundance and greater microbial diversity, changes that may have supported improved prepartum body condition score through enhanced fermentative capacity and metabolic efficiency. These maternal alterations appeared to influence neonatal gut colonization, as calves born to BAC-supplemented heifers exhibited similar microbiota shifts despite the absence of direct DFM supplementation. Such transgenerational effects suggest that fecal microbiome changes during the periparturient and early postnatal periods may have contributed to improved calf growth and immune function. Collectively, these results highlight maternal *Bacillus*-based DFM supplementation as a promising strategy to beneficially modulate both maternal and offspring microbiota, thereby supporting long-term productivity through transgenerational microbial programming.
